# ESBL-producing *Klebsiella pneumoniae* gut colonisation and subsequent health-care associated bacteraemia in preterm newborns: a descriptive cohort with nested case–control study

**DOI:** 10.1017/S0950268825100630

**Published:** 2025-10-06

**Authors:** Moussa Benboubker, Bouchra Oumokhtar, Driss Oukachou, Samira Elfakir, Salim Belchkar, Manal Rossi, Abdelhamid Massik, Ghita Yahyaoui, Kaoutar Moutaouakkil, Fouzia Hmami

**Affiliations:** 1 Human Pathology Biomedicine and Environment Laboratory, Faculty of Medicine and Pharmacy, Sidi Mohammed Ben Abdellah University, Fez, Morocco; 2 Department of Neonatology and Neonatal Intensive Care, Faculty of Medicine and Pharmacy, Sidi Mohammed Ben Abdellah University, Fez, Morocco; 3 Department of Epidemiology and Public Health, Faculty of Medicine and Pharmacy, Sidi Mohammed Ben Abdellah University, Fez, Morocco; 4 Epidemiology and Health Science Research Laboratory, Faculty of Medicine and Pharmacy, Sidi Mohammed Ben Abdellah University, Fez, Morocco; 5 Biomedical and Translational Research Laboratory, Faculty of Medicine and Pharmacy, Sidi Mohamed Ben Abdellah University, Fez, Morocco; 6 Laboratory of Microbiology and Molecular Biology, Faculty of Medicine and Pharmacy, Sidi Mohammed Ben Abdellah University, Fez, Morocco

**Keywords:** antibiotic resistance, ESBL-producing *Klebsiella pneumoniae*, healthcare-associated bacteraemia, intestinal colonisation, preterm

## Abstract

This descriptive and exploratory observational case series examined intestinal colonisation and subsequent bacteraemia due to ESBL-producing *Klebsiella pneumoniae* (ESBL-*Kp*) in preterm neonates in Morocco. Prospective bacteriological cultures and antibiotic susceptibility testing were supported by phenotypic methods, including Brilliance ESBL Agar and the NG-Test CARBA-5 assay, for the rapid detection of ESBL and carbapenemase producers. Molecular analysis using PCR was also undertaken to identify specific resistance genes. A total of 567 rectal swabs were collected from 339 preterm neonates, yielding 293 *K. pneumoniae* isolates. ESBL-producing strains were identified in 53.6% of the neonates (182/339). Detected resistance genes included *bla*SHV (26.3%), *bla*CTX-M-1 (42.8%), *bla*TEM (30.2%), *bla*OXA-48 (50.0%), *bla*NDM(15.3%), and *bla*VIM (4.9%). Principal risk factors for colonisation were low birth weight (OR 1.69), very preterm birth (OR 6.24), enteral tube feeding (OR 2.02), and prolonged use of third-generation cephalosporins (OR 1.26). Among the neonates studied, 32 (9.4%) developed healthcare-associated bacteraemia, with 56.2% of these cases preceded by intestinal colonisation with ESBL-*Kp.* Clinically, severe respiratory distress and alveolar haemorrhage were strongly associated with increased mortality (aRR = 29.32 and 4.45, respectively). The findings highlight the clinical importance of early screening to guide infection control and antimicrobial stewardship in neonatal intensive care settings.

## Introduction

Healthcare-associated bacteraemia in preterm neonates presents a major challenge in neonatal intensive care units (NICUs), particularly in low- and middle-income countries (LMICs), where access to effective antimicrobial therapies remains limited [1]. Preterm neonates, born before 37 weeks of gestation, are at higher risk owing to their immature immune systems and frequent exposure to invasive medical procedures. Bacteraemia, defined as the presence of bacteria in the bloodstream, can lead to severe complications such as sepsis, organ dysfunction, prolonged hospital stays, and neonatal mortality. In addition, the long-term developmental consequences of sepsis include neurological deficits and developmental delays [2, 3].

In recent years, the emergence and spread of extended-spectrum β-lactamase-producing *K. pneumoniae* (ESBL*-Kp)* have been widely documented in healthcare settings, particularly in neonatal units [4]. In LMICs, the incidence of neonatal *K. pneumoniae* infections ranges from 4.1 to 6.3 per 1,000 live births, with a case fatality rate of 18% to 68% [2, 3]. *K. pneumoniae* accounts for 16% to 28% of confirmed neonatal sepsis cases based on blood cultures in various countries [5]. Bacteraemia in preterm neonates can occur when gut bacteria, such as *K. pneumoniae*, translocate into the bloodstream, facilitated by disruptions in the gut barrier, bacterial translocation, and a weakened immune response [6, 7]. The intestinal carriage of ESBL*-Kp* is a major concern, as it can serve as a reservoir for future infections. Contributing factors include broad-spectrum antibiotic use, prolonged hospitalisation, invasive procedures, and the immaturity of the neonatal immune system [8]. Colonisation can occur through contact with healthcare workers, contaminated medical equipment, or other colonised patients [9].

The intestinal colonisation of ESBL*-Kp* can persist for extended periods in preterm neonates, and it serves as a potential source of infections, primarily through the ascension from the gut into the bloodstream, leading to sepsis, meningitis, and other serious conditions [10]. Infections caused by ESBL-pneumoniae are associated with limited therapeutic options, leading to increased mortality and prolonged hospital stays [11]. Furthermore, preterm infants who acquire ESBL*-Kp* infection face potential long-term complications and consequences, including neurodevelopmental outcomes, respiratory sequelae, and impacts on growth and nutrition [12].

Understanding the dynamics of intestinal carriage and subsequent infection with ESBL-*Kp*, particularly bloodstream infections in preterm neonates, is crucial for effective infection control in the NICU. Key preventive measures include strict hand hygiene, cohorting of colonised patients, and antimicrobial stewardship to limit the spread and impact of ESBL-*Kp* in this vulnerable population [13].

The primary objective of this study was to assess the prevalence of intestinal colonisation by ESBL-producing *K. pneumoniae* in a cohort of 339 preterm newborns, and to investigate potential risk factors for subsequent bacteraemia through a nested case–control analysis of 32 cases.

## Materials and methods

### Study design and patient cohort

This descriptive cohort, with a nested case–control analysis was conducted over 3 years (November 2020 to November 2023) in the neonatal intensive care unit (NICU) of a Moroccan hospital. The study included 32 preterm neonates who developed healthcare-associated bacteraemia (HAB) and aimed to document the prevalence of intestinal colonisation by ESBL-producing *K. pneumoniae* (ESBL-*Kp*) and subsequent HAB, without implying causality. Infants born at ≥37 weeks, died or discharged/dying within 48 h were excluded. Active rectal surveillance was performed at admission, on days 5 and 10, and at regular intervals during prolonged hospitalisation, yielding 567 rectal swabs from 339 preterm neonates and 293 *K. pneumoniae* isolates. Thirty-two clinical strains were recovered from blood cultures of neonates with HAB.

### Study outcomes and statistical analysis

For exploratory analyses, colonised neonates were considered “exposed” and non-colonised “non-exposed,” with HAB as the primary outcome. Time zero was defined as the first available screening, and follow-up ended at HAB onset, death, or discharge. Death and live discharge were treated as competing events. Kaplan–Meier survival curves and Fine–Grey cumulative incidence models were applied, along with cause-specific Cox regression, to evaluate associations between exposures and outcomes.

A nested case–control analysis compared colonised neonates who developed HAB (cases) to HAB patients without prior ESBL-*Kp* colonisation (controls) to identify potential risk factors. Continuous variables were analysed using Student’s *t*-test, and categorical variables using chi-square or Fisher’s exact test. Significant variables were included in a logistic regression model with stepwise backward selection (entry *P* = 0.05, removal *P* = 0.10) to identify independent factors associated with ESBL-*Kp* colonisation, reporting adjusted odds ratios (ORs) and 95% confidence intervals (CIs), controlling for confounders such as sex, birth weight, mode of delivery, gestational age, duration of hospitalisation, and history of PPROM. Time-to-event analyses also assessed colonisation and HAB onset under different antibiotic exposures (third-generation cephalosporins and carbapenems).

Analyses were conducted using SPSS version 23 and R version 3.4.0, with two-sided *P*-values <0.05 considered statistically significant. The study protocol was approved by the Ethics Committee of the Faculty of Medicine and Pharmacy, Hassan II University Hospital, Fez, Morocco, and informed consent was obtained from parents or legal guardians.

### Sample size

The sample size estimation was based on several assumptions derived from the literature and preliminary data [14]. The expected prevalence of intestinal colonisation with extended-spectrum β-lactamase–producing Enterobacteriaceae (ESBL-E) among preterm neonates admitted to the neonatal unit was estimated at 50%–60%. Among colonised infants, approximately 10% were expected to progress to sepsis, compared with about 4% in non-colonised infants, corresponding to an anticipated relative risk of 2.5. Statistical parameters included a significance level of 5% (type I error) and a statistical power of 80% (type II error set at 20%).

The approach adopted was that of a prospective observational study comparing two groups (exposed vs. non-exposed). The calculation of the sample size was based on the classical formula for comparing two independent proportions [15]:

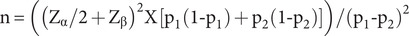

where *Z*_α/2_ = 1.96, corresponding to a 95% confidence interval, Z_β_ = 0.84, corresponding to a statistical power of 80%, *p*_1_ = 0.10, representing the estimated progression to sepsis among colonised neonates, *p*_2_ = 0.04, representing the estimated progression to sepsis among non-colonised neonates.

### Identification, phenotyping, and antimicrobial susceptibility testing

All isolates were identified based on microscopic, morphological, and biochemical characteristics. Cultures were initiated on Eosin Methylene Blue (EMB) agar, and strain identification was performed using the API E20 system (BioMérieux). Antimicrobial susceptibility was assessed against 17 agents, including imipenem, meropenem, ertapenem, amoxicillin–clavulanic acid, ticarcillin–clavulanic acid, piperacillin–tazobactam, ceftazidime, cefoxitin, cefotaxime, amikacin, cefepime, gentamicin, levofloxacin, ciprofloxacin, nalidixic acid, trimethoprim–sulfamethoxazole, and fosfomycin.

Extended-spectrum β-lactamase (ESBL) production was screened using Brilliance ESBL Agar (Oxoid). Carbapenemase production (KPC, OXA-48, VIM, IMP, NDM) was detected qualitatively using the NG-Test CARBA-5 (NG Biotech), with results incorporated as qualitative variables. Phenotypic data were correlated with the presence of corresponding resistance genes (*bla*KPC, *bla*OXA-48, *bla*VIM, *bla*IMP, *bla*NDM) to assess concordance. *Escherichia coli* ATCC® 25922 was used as a control, and results were interpreted according to EUCAST guidelines.

### Molecular analysis of ESBL-producing strains

Only isolates from colonised patients who developed ESBL-*Kp* bacteraemia underwent molecular analysis. Polymerase chain reaction (PCR) assays were performed to detect β-lactamase-encoding genes, including *bla*CTX-M (phylogenetic groups 1, 2, and 9), *bla*TEM, *bla*SHV, and carbapenemase genes *bla*KPC, *bla*NDM, *bla*VIM, and *bla*OXA-48, following previously described protocols. [16]

### Study definitions

**Neonatal bacteraemia [or bloodstream infection]** was defined according to the criteria of the Centers for Disease Control and Prevention (CDC) [17].

**Late-onset sepsis.** Sepsis is defined as a life-threatening organ dysfunction caused by a dysregulated host response to infection. In neonates, late-onset sepsis (LOS) is distinguished from early-onset sepsis (EOS), which occurs within the first 48–72 h after birth. LOS is specifically defined as sepsis that manifests after 72 h post-delivery and can extend up to 28 days corrected gestational age [18, 19].

**Hospital-acquired bacteraemia.** Bacteraemia was classified as community-acquired bacteraemia (CAB) or hospital-acquired bacteraemia (HAB). HAB was defined as a positive blood culture infection acquired after at least 48 h of hospital admission. HAB also included cases in which patients were transferred to our hospital from other hospitals where they had been hospitalised for over 48 h and the blood culture collected within 48 h of transfer was positive. All remaining cases were classified as CAB [20].

**Adverse neonatal outcomes.** These are defined as any significant health issues occurring during the neonatal period. Key indicators include low birth weight (LBW), preterm delivery, Apgar scores, neonatal death, small for gestational age (SGA), and severe neonatal conditions such as respiratory distress syndrome, infections, or other significant morbidities [21].

**Prolonged exposure to third-generation cephalosporins (C3G).** We defined prolonged exposure to third-generation cephalosporins (C3G) as the continuous administration of a C3G antibiotic (e.g., cefotaxime, ceftazidime, or ceftriaxone) for a duration of five or more consecutive days during hospitalisation in the neonatal intensive care unit (NICU) [22].

## Results

### Patient cohort and risk factors for ESBL-*Kp*

Out of 339 preterm neonates, 182 (53.6%) tested positive for intestinal colonisation with ESBL-producing *K. pneumoniae* (ESBL-*Kp*), of which 26.8% carried KPC-producing strains during active rectal screening. Factors significantly associated with colonisation included low birth weight (OR = 1.69; *p* = 0.046), very preterm status (OR = 6.24; *p* = 0.038), enteral tube feeding (OR = 2.02; *p* = 0.046), and prolonged exposure to third-generation cephalosporins (OR = 1.26; *p* = 0.001), as identified in both univariate and multivariate analyses (Table 1).

**Table 1.** Preterm newborns baseline clinical characteristics and logistic regression of ESBL*-Kp* carriage status

Variable	ESBL*-Kp* colonisation status		Univariate analysis		Multivariate analysis	
	Yes (*N* = 182, 53.6%)	No (*N* = 157, 46.3%)	OR (95% CI)	*P*	OR (95% CI)	*p*
Sex (Female)^a^	84 (46.2)	75 (47.8)	0.93(0.61–1.43)	0.766		
Sex (Male)^a^	98 (53.8)	82 (52.2)	1	-		
Birth Weight (g)^b^	1844:955	1840: 628	-	0.963	1.69(1.00–2.83)	0.046
Delivery Mode (Caesarean birth)^a^	84 (46.2)	54 (34.4)	1.63(1.05–2.53)	0.028		
Delivery Mode (Vaginal birth)^a^	98 (53.8)	103 (65.6)	1	-		
Prematurity categories						
Very preterm^a^	57(31.3)	42(26.8)	4.07(0.78–21.18)	0.095	6.24(1.09–35.46)	0.038
Moderate and late preterm^a^	123(67.6)	109(69.4)	3.38(0.66–17.12)	0.140		
Extremely preterm^a^	2(01.0)	6(03.8)	1	-		
Associated pathology						
Respiratory distress^a^	162(89.0)	132(84.1)	1.53(0.81–2.88)	0.183		
Jaundice and /or Neonatal suffering^a^	22(12.0)	42(26.8)	0.37(0.21–0.66)	0.000	0.37(0.19–0.72)	0.003
Invasive procedures						
Peripheral intravenous catheter^a^	180(98.9)	147(93.6)	6.12(1.32–28.38)	0.020		
Umbilical vein catheter^a^	118(64.8)	76(48.4)	1.96(01.27–3.04)	0.002		
Enteral tube feeding^a^	152(83.5)	107(68.2)	2.36(1.41–3.96)	<0.001	2.02(1.01–4.07)	0.046
Antimicrobial exposure duration (Days)						
Cephalosporines 3rd Generation^b^	3.08:2.62	1.73:2.70	1.22(1.11–1.33)	<0.001	1.26(1.13–1.41)	<0.001
Aminopenicillins^b^	2.47:2.53	1.89:2.32	1.10(1.01–1.20)	0.029		
Aminoglycosides^b^	3.35:2.47	2.64:2.06	1.14(1.04–1.26)	0.005		
Outcomes						
Length of stay (Day)^c^	7(01–42)	6(01–51)	1.01(0.98–1.03)	0.444	0.96(0.93–1.00)	0.070
BSI (ESBL*-Kp*)^a^	18(09.8)	14(08.9)	0.74(0.36–1.51)	0.415		
Dead^a^	36(19.7)	36(22.9)	1.20 (0.71–2.03)	0.479		

Abbreviations: OR, odds ratio; CI, confidence interval; BSI, Bloodstream Infection.

a Frequency [%].

b Mean [SD].

c Median [range].

**Figure 1.**
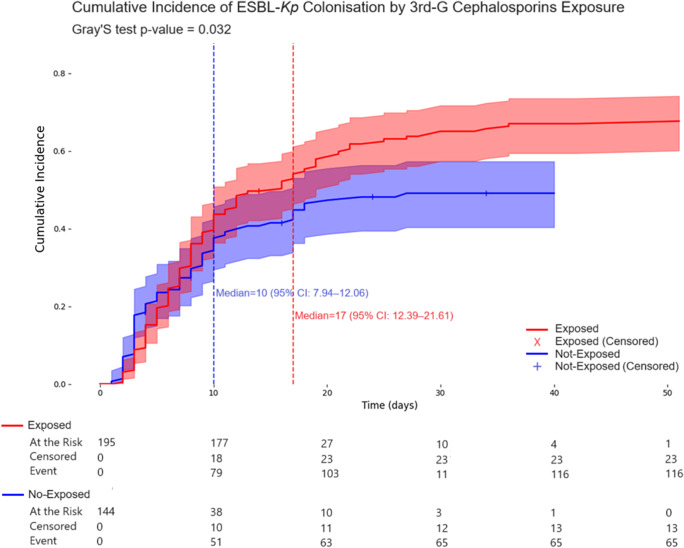
The cumulative incidence curve illustrates the comparison of days of ESBL*-Kp* colonisation between the groups exposed and not exposed to third-generation cephalosporins. The median differs for both groups at the onset of antibiotic therapy.

**Figure 2.**
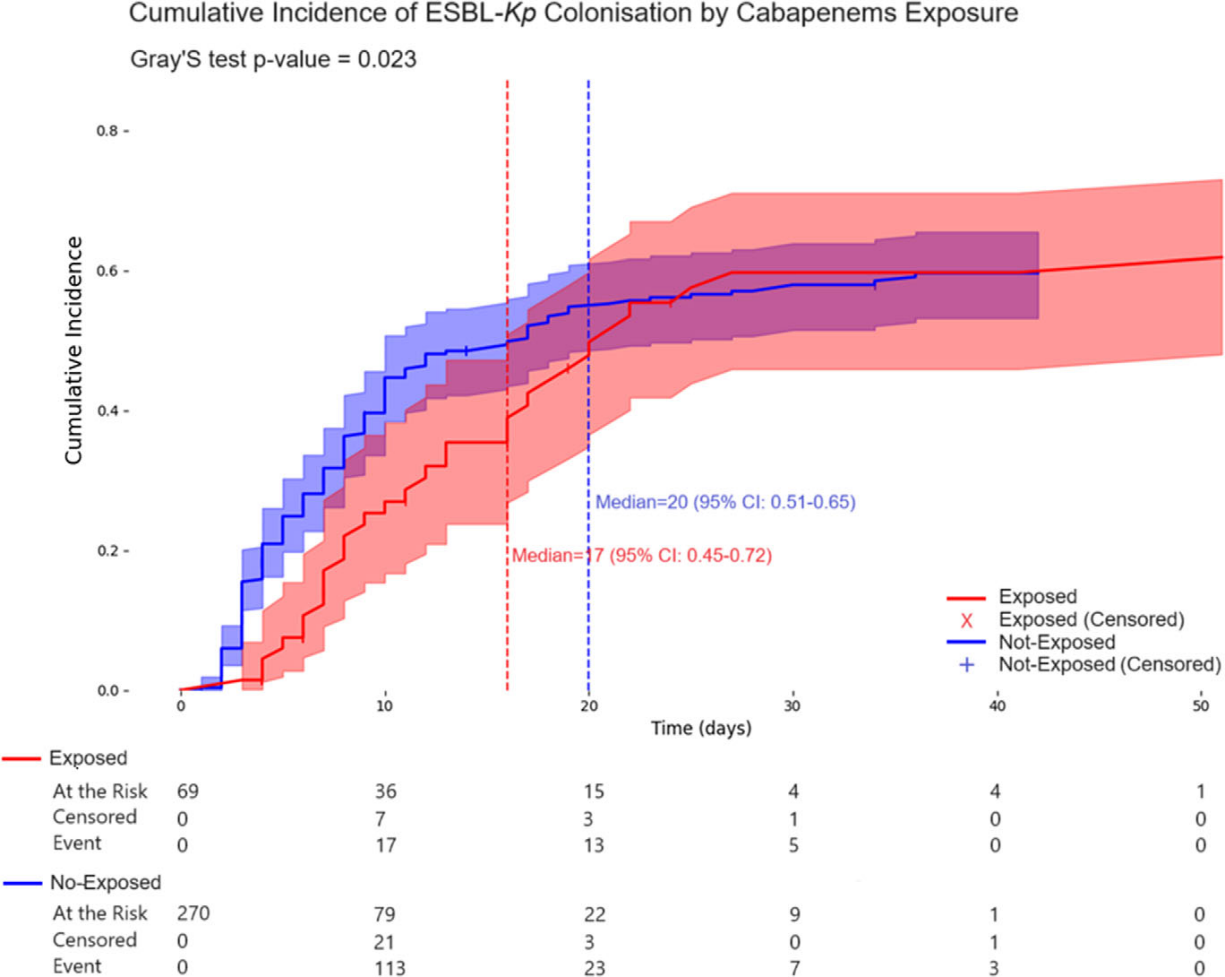
The cumulative incidence curve illustrates the comparison of days of ESBL-*Kp* colonisation between the groups exposed and not exposed to carbapenems. The median differs for both groups at the onset of antibiotic therapy.

Exposure to broad-spectrum antibiotics was also associated with an increased cumulative incidence of ESBL-*Kp* intestinal colonisation. Kaplan–Meier cumulative incidence curves showed that neonates exposed to third-generation cephalosporins had a significantly higher risk of colonisation compared with unexposed neonates (Grey’s test, *p* = 0.032), with a median time to colonisation of 10 days (95% CI: 7.94–12.06) versus 17 days (95% CI: 12.39–21.61) in the non-exposed group (Figure 1). Similarly, carbapenem exposure was associated with earlier colonisation (Grey’s test, *p* = 0.023), with a median time to colonisation of 17 days (95% CI: 0.45–0.72) compared to 20 days (95% CI: 0.51–0.65) in non-exposed neonates (Figure 2).

Overall, the incidence of ESBL-*Kp* healthcare-associated bacteraemia (HAB) was 9.4% (32/339). Among colonised neonates, 18/182 (9.8%) developed HABs, versus 14/157 (8.9%) in the non-colonised group, indicating comparable occurrence of HABs regardless of prior intestinal carriage (Tables 1 and 3).

### Antimicrobial susceptibility and genetic diversity

During rectal screening, 293 non-duplicate *K. pneumoniae* isolates were recovered. Of these, 182 isolates (62.1%) were ESBL producers, with 50% carrying KPC carbapenemases. High resistance rates were observed for ertapenem (50.0%), piperacillin-tazobactam (100%), ticarcillin-clavulanic acid (65.9%), amoxicillin-clavulanic acid (92.8%), ceftazidime (67.5%), cefepime (60.9%), cefotaxime (56.0%), and levofloxacin (69.7%), while lower resistance rates were seen for cefoxitin (26.9%), imipenem (18.2%), and meropenem (12.0%). Resistance genes included *bla*CTX-M1 (98.3%), *bla*TEM (30.2%), and *bla*SHV (26.3%), with carbapenemase genes *bla*OXA-48 (50.0%), *bla*NDM (15.3%), and *bla*VIM (4.9%) detected in subsets of isolates (Table 2).

**Table 2.** Resistance patterns and genetic profile of ESBL*-Kp* isolates from Blood culture and intestinal colonisation

	ESBL-Kp Isolates	
	Intestinal colonisation (*n* = 182, 53.6%)	Blood culture (*n* = 32, 9.4%)
**Antimicrobial agents**		
Imipenem (10 μg)	33(18.1%)	2(6.2%)
Meropenem (10 μg)	22(12%)	1(3.1%)
Ertapenem (10 μg)	91(50%)	2(6.2%)
Piperacillin-tazobactam (30–6 μg)	182 (100%)	32(100%)
Ticarcillin-clavulanic acid (75 μg)	120(65.9%)	32(100%)
Amoxicillin-clavulanic acid (30 μg)	169(92.8%)	32(100%)
Ceftazidime (30 μg)	123 (67.5%)	32(100%)
Cefoxitin (30 μg)	111 (60.9%)	15(46.8%)
Cefepime (30 μg)	49 (26.9%)	—
Cefotaxime (30 μg)	102 (56%)	32(100%)
Amikacin (30 μg)	99(54.3%)	30(93.7%)
Gentamicin (10 μg)	182 (100%)	32(100%)
Levofloxacin (5 μg)	127(69.7%)	18(50%)
Nalidixic acid (30 μg)	73(40.1%)	17(53.1%)
Trimethoprim/ sulfamethoxazole (25 μg)	103 (56.5%)	28 (43.3%)
**Genetic profile**		
*bla*OXA–48	91(50%)	1(3.1%)
*bla*NDM	28(15.3%)	1(3.1%)
*bla*VIM	9(4.9%)	—
*bla*KPC	2(1%)	—
*bla*CTXM–1	179(98.3%)	32(100%)
*bla*CTXM–2	10(5.4%)	—
*bla*CTXM–9	8(4.3%)	—
*bla*SHV	48(26.3%)	32(100%)
*bla*TEM	55(30.2%)	21 (69.4%)

Abbreviations: VIM, verona integron-encoded metallo-β-lactamase; NDM, New Delhi metallo-β-lactamase; KPC, *K. pneumoniae* carbapenemase; OXA-48, oxacillinase 48; CTX-M1–2-9, cefotaximase-Munich; SHV, sulphhydryl variable; TEM, temonera.

Among 32 ESBL-*Kp* blood isolates from HABs, all were resistant to piperacillin-tazobactam, ticarcillin-clavulanic acid, amoxicillin-clavulanic acid, ceftazidime, and cefotaxime, while resistance to imipenem (6.2%), meropenem (3.1%), and ertapenem (6.2%) was lower. Molecular analysis showed universal presence of blaSHV and blaCTX-M1 (100%), and high prevalence of *bla*TEM (69.4%). Two isolates carried carbapenemase genes (*bla*OXA-48 and *bla*NDM) (Table 2). Biotyping based on antibiotic susceptibility and resistance gene patterns revealed three distinct clusters. Cluster 1 (Top-Left) predominantly harboured *bla*CTX-M9, *bla*TEM, and *bla*SHV, and exhibited notable carbapenem resistance (73% similarity; Euclidean distance 2.83). Cluster 2 (Middle) displayed mixed susceptibility profiles associated with *bla*CTX-M1 and *bla*SHV, with limited carbapenem resistance (67% dissimilarity; Euclidean distance 3.32). Cluster 3 (Bottom) showed extensive resistance and was strongly associated with *bla*OXA-48 and Carba-NG positivity, suggesting probable carbapenemase production (70% dissimilarity; Euclidean distance 2.65) (Supplementary Figure A1).

### Clinical outcomes and mortality of ESBL-*Kp* HABs

Among the 32 HAB cases, 18 neonates (56.2%) had positive intestinal carriage (case group), while 14 (43.7%) were non-colonised (control group). Both groups included four extremely preterm infants evenly distributed (2 per group). The colonised group was predominantly female (55.5%) and had median postnatal age 9 h, gestational age 34 weeks, birth weight 1,560 g, and Apgar scores of 9 and 10 at 1 and 5 min, respectively. Caesarean deliveries accounted for 16.7% (Table 3 and Supplementary Table A1).

**Table 3.** Clinical characteristics, serum markers, and complications of preterm infants with HABs ESBL*-Kp* stratified by Colonisation status

	Case group (*N* = 18)	Control group (*N* = 14)	*P*
Demographic characteristics			
Sex/Female	10(55.5%)	05(35.7%)	0.307
PPA (hours)	09(1.00–36.00)	27(5.00–72.00)	0.046
GA (wk)	34(27.75–36.00)	35(30.00–36.00)	0.411
BBW(g)	1,560(100–2,350)	2,300(1150–3,100)	0.012
C/S	03(16.6%)	-	0.164
Apgar score 1	9(6–10)	10(7–10)	0.722
Apgar score 5	10(8–10)	10(9–10)	0.649
Serum markers			
CRP (mg/L)	77(29.00–91.50)	79(77.00–80.75)	0.009
WBC (10^3^/μl)	15.46(08.86–23.00)	16.90(07.96–17.78)	0.091
Hgb (g/dl)	11.40(0–16)	16.50(0–18.10)	0.114
PLT (10^3^/μl)	217(130–237.50)	201(12.50–299)	0.011
Antimicrobial therapy^a^			
Tienam (25 mg/kg/12 h)	10(55.5%)	7(50%)	0.517
Ciprofloxacin (15 mg/kg/12 h)	9(50%)	4(28.5%)	0.195
Amiklin (15 mg/kg/24 h)	12(66.6%)	10(71.4%)	0.540
Triaxon (100 mg/kg/24 h)	13(72.2%)	12(85.7%)	0.318
Gentamicin (04 mg/kg/24 h)	14(77.7%)	12(85.7%)	0.460
Colymicin (50,000 UI/kg/08 h)	1(05.5%)	2(14.2%)	0.403
Vancomycin (25 mg/kg/24 h)	5(27.7%)	1(7.1%)	0.152
Clinical Complications and Outcomes			
Cerebral Haemorrhage	3(16.6%)	-	0.164
Active Alveolar Haemorrhage	6(33.3%)	2(4.2%)	0.207
Renal Failure	3(16.6%)	-	0.237
Sepsis And Acute Circulatory Failure	2(11.1%)	1(7.1%)	0.596
Aggravation of Respiratory Distress	2(11.1%)	6(42.8%)	0.049
Pneumothorax	1(5.5%)	2(14.2%)	0.403
Enterocolitis	2(11.1%)	-	0.308
Mortality rate	12(66.6%)	11(78.5%)	0.367

*Note:* All values are shown as n [%] or median [interquartile range, 25%–75%].

Abbreviations: Apgar score1 = Apgar score at 1 min; Apgar score 5 = Apgar score at 5 min; BBW = Birth Body Weight; C/S = Caesarean Section; CRP = C-Reactive Protein; GA = Gestational Age; Hgb = Haemoglobin; PLT = Platelet; PPA = Postpartum Age; WBC = White Blood Count.

a Antimicrobial therapy protocol is detailed in Supplementary Table A2.

Significant differences between colonised and non-colonised neonates were observed in postnatal age and birth weight, whereas sex, gestational age, mode of delivery, and Apgar scores were comparable. Colonised infants had higher platelet counts (217 × 10^6^/μL vs. 201 × 10^6^/μL; *p* < 0.05) and lower CRP levels (77 vs. 179; *p* < 0.05). Differences in WBC and haemoglobin were not statistically significant (WBC: p = 0.09; Hgb: *p* = 0.114).

Clinical complications were similar overall, except for worsening respiratory distress, which was less frequent among colonised infants (11.1%) compared with non-colonised infants (42.8%; p < 0.05). No differences were observed in cerebral haemorrhage, alveolar haemorrhage, renal failure, sepsis, circulatory failure, or antibiotic treatment (Table 3 and Supplementary Table A1).

The overall mortality rate was 21.2% (72/339), with no significant difference between colonised and non-colonised neonates (Table 1). HAB-associated mortality was higher (71.8%; 23/32), but remained comparable between colonised (66.7%) and non-colonised neonates (78.5%; *p* = 0.367; Table 3). Kaplan–Meier survival analyses showed no significant difference in mortality according to colonisation (log-rank *p* = 0.497) or bacteraemia (log-rank *p* = 0.330), with median survival times of 34 and 10 days, respectively (95% CI: 0.43–0.67 for colonisation; 0.13–0.70 for bacteraemia), and overlapping confidence intervals and nearly superimposed survival curves.

**Figure 3.**
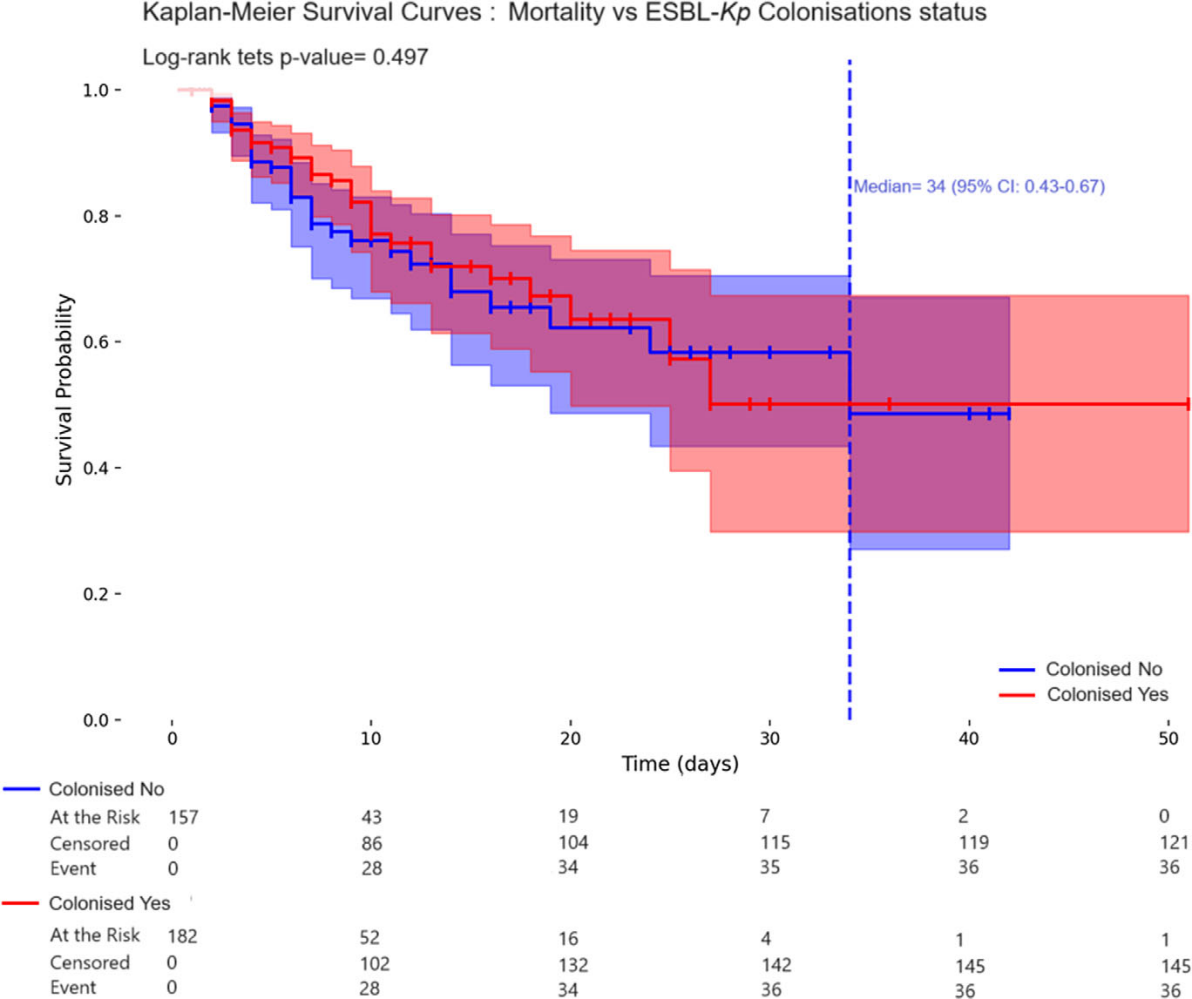
Kaplan–Meier survival estimates among preterm neonates by ESBL*-Kp* colonisation status. The median survival time appears to be approximately the same for both groups.

**Figure 4.**
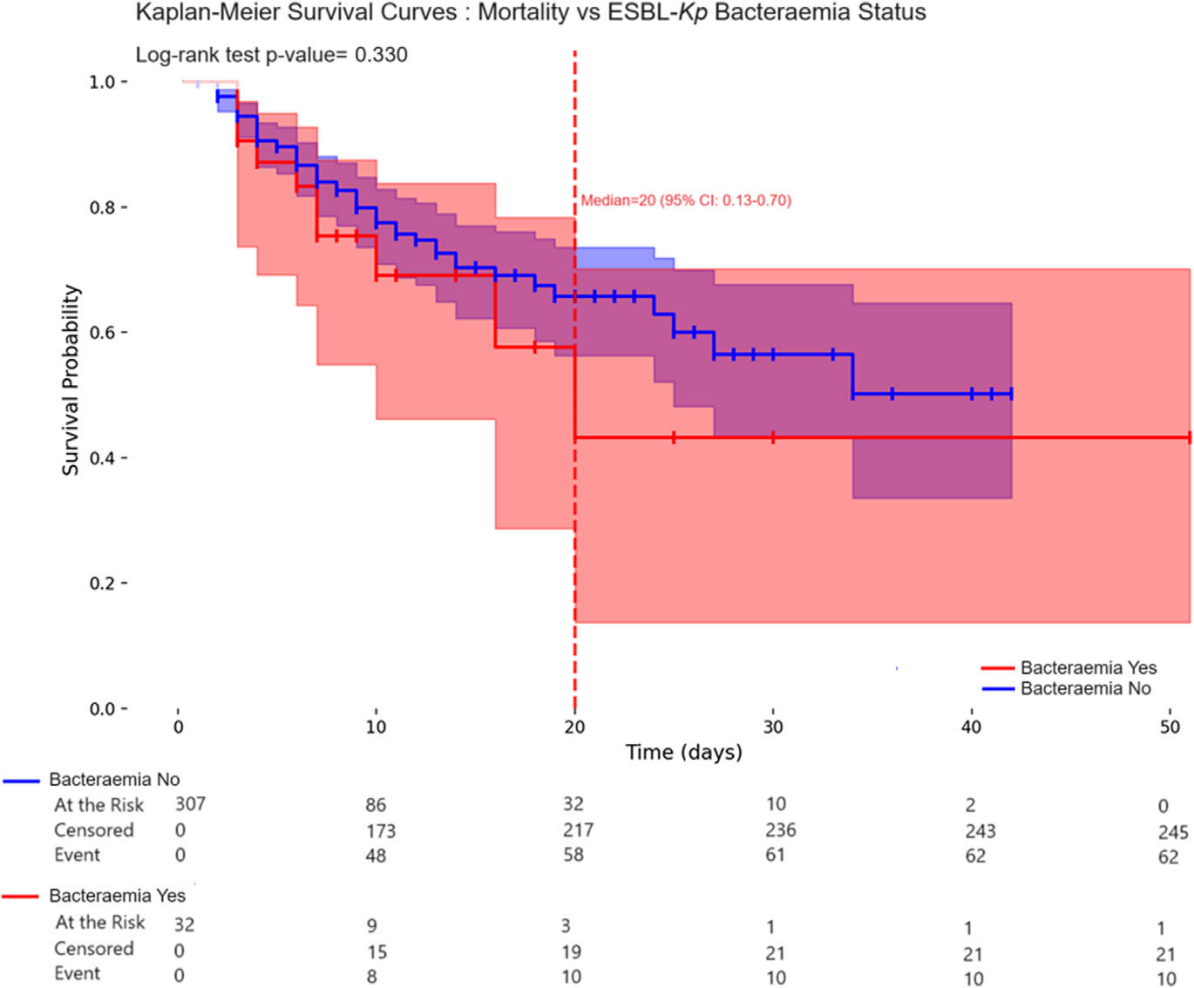
Kaplan–Meier survival estimates among preterm neonates with and without ESBL*-Kp* HABs.

Multivariate analysis identified worsening respiratory distress (aRR = 29.32; 95% CI: 2.66–322.37; *p* = 0.005) and alveolar haemorrhage (aRR = 4.45; 95% CI: 1.03–19.10; *p* = 0.044) as independent predictors of increased mortality. In contrast, prematurity and the presence of HABs were not significantly associated with mortality outcomes (Table 4 and Figures 3 and 4).

**Table 4.** Survival with HABs, stratified by prematurity categories and clinical complications

	aRR (95% CI)	*P*
Prematurity categories		
Very preterm	0.71(0.07–6.40)^a^	0.762
Moderate and late preterm	0.87 (0.137–5.52)^a^	0.882
Clinical complications		
Aggravation of respiratory distress	29.32(2.66–322.37)^a^	0.005
Alveolar haemorrhage	4.45(1.03–19.10)^a^	0.044
Sepsis and acute circulatory failure	4.98(0.61–40.42)^a^	0.132

a Adjusted for GA, Vaginal Delivery, and Sex.

## Discussion

Over a 3-year period, we monitored intestinal acquisition of ESBL-*Kp* in preterm infants hospitalised in the NCUI, aiming to investigate the widely debated link between gut colonisation and subsequent ESBL-*Kp* bacteremia. Among the 339 preterm infants included, 182 (53.6%) were colonised during hospitalisation according to our active screening protocol. This high prevalence is consistent with previous studies on gut colonisation [23, 24]. Notably, gastrointestinal colonisation with *K. pneumoniae* is common in neonates, with reported rates as high as 87%, and up to 70% of infections linked to prior colonisation in NICU patients [14].

In our cohort, enteral tube feeding was associated with ESBL-*Kp* colonisation, corroborating findings by Petersen et al., who showed that enteral tubes increase bacterial load from day one [25]. Additional studies have demonstrated that microbial flora in feeding tubes contributes to intestinal colonisation in neonates [26]. Moreover, prolonged exposure to third-generation cephalosporins was significantly associated with ESBL-*Kp* acquisition. This aligns with findings by Tacconelli et al. and Rohde et al., who identified empirical antibiotic treatment as a major risk factor for colonisation in neonates [27, 28]. While some research suggests early antibiotic use does not increase colonisation by multidrug-resistant organisms [29], other studies, including ours, support its role as an independent risk factor. The effect varies depending on whether antibiotics are used as monotherapy or in combination [27].

We estimated the overall prevalence of healthcare-associated bacteremia (HAB) due to ESBL-*Kp* in our cohort at 9.43% (32/339). Among colonised infants, 18 of 182 (9.8%) developed HAB, compared to 14 of 157 (8.9%) in the non-colonised group – rates similar to those reported elsewhere. Chen et al. found a bacteremia incidence of 8.0% in colonised neonates versus 1.9% in non-colonised ones [30]. Their homology analysis also demonstrated close genetic relatedness between intestinal and extraintestinal isolates, supporting the hypothesis that the gut serves as a reservoir for systemic infections [30].

Our molecular analysis showed that gut colonisation isolates carried a high prevalence of *bla*CTX-M-1(98.3%), with moderate rates of *bla*SHV (26.37%) and *bla*TEM (30.2%). The detection of carbapenemase genes, *bla*OXA-48 (50.0%), *bla*NDM (15.3%), and *bla*VIM (4.9%) indicates the presence of multidrug-resistant strains. Among bacteremia isolates, we observed 100% prevalence of *bla*CTX-M-1, and *bla*TEM (69.4%), with sporadic detection of *bla*OXA-48, and *bla*NDM in two cases. This variation reflects the capacity of *K. pneumoniae* to acquire resistance genes under antibiotic pressure [31].

In our study, we utilised antibiotic susceptibility testing, phenotyping, and genetic diversity analysis as biotyping tools to evaluate the similarities. Our results support the clonal relationship hypothesis, suggesting that bacteremia frequently originates from intestinal strains [14]. Notably, *K. pneumoniae* displayed broad resistance, particularly to cephalosporins. Third-generation cephalosporins, especially ceftriaxone, were commonly used in the NICU for empirical treatment of clinical sepsis, often alongside aminoglycosides. We hypothesize that this selective pressure contributed to the emergence of resistant strains [32, 28].

C-reactive protein (CRP) remains the standard serum marker for neonatal bacteremia, but distinguishing between postnatal physiological elevation and infection remains difficult [33]. In our study, the CRP cut-off for late-onset sepsis (LONS) due to ESBL-*Kp* showed a significant difference between colonised and non-colonised infants: 77 (29.00–91.50) vs. 79 (77.00–80.75), *p* < 0.05. This contradicts our hypothesis that colonised infants would have higher CRP levels. Although elevated CRP may reflect increased bacterial translocation in certain conditions, such as bowel obstruction, a single CRP value is not a reliable diagnostic marker for HAB [34]. Serial CRP measurements at 6–12 h after onset are recommended for better diagnostic accuracy.

Clinical complications significantly affected mortality. Worsening respiratory distress and alveolar haemorrhage were associated with increased risk of death (aRR = 29.32, 95% CI = 2.66–322.37, *p* = 0.005; and aRR = 4.45, 95% CI = 1.03–19.10, *p* = 0.044, respectively). In contrast, gestational age, sepsis category, birth weight, and Apgar scores were not significantly associated with mortality, consistent with other studies [35].

The overall mortality rate in our cohort was 21.2% (72/339), with no significant difference between colonised and non-colonised groups. Among HAB cases, mortality reached 71.8% (23 cases), with similar rates in colonised (66.6%, 12/18) and non-colonised (78.5%, 11/14) infants. These results align with prior findings, including a 28.6% mortality rate in preterm infants reported by Lemma et al., especially during the first week of life [36]. Mortality has also been correlated with lower gestational age, low birth weight, and respiratory distress syndrome, which accounts for up to 45% of neonatal deaths [37, 38]. In our study, only three colonised infants with HAB presented with acute circulatory failure, without significant group differences.

This study has several limitations. First, the absence of complete homology data between intestinal and bloodstream isolates restricts our capacity to confirm direct transmission pathways. Second, the sample size estimation, although based on established formulae and adjusted for an anticipated 10% rate of missing data, was not fully achieved, thereby limiting the statistical power and reducing the strength of causal inferences. Third, surveillance bias may have occurred, as neonates with longer hospital stays or greater exposure to medical interventions were subjected to more frequent cultures, potentially increasing the likelihood of detecting transmission. Accordingly, the findings should be considered exploratory and primarily hypothesis-generating, highlighting the need for larger, multicentre cohort studies in the future.

## Conclusion

While no significant difference in mortality was found between colonised and non-colonised groups with HAB, our findings underscore the clinical importance of intestinal ESBL-*Kp* colonisation in preterm infants. HAB due to ESBL-*Kp* is associated with high morbidity and mortality and highlights the urgent need for infection control measures in NICUs. The potential for clonal dissemination within hospital settings necessitates strict hygiene protocols, early screening, and containment of colonised cases to reduce the risk of life-threatening infections in this vulnerable population.

## Supporting information

10.1017/S0950268825100630.sm001 Benboubker et al. supplementary material Benboubker et al. supplementary material

## Data Availability

The datasets supporting this study are included in the article, and excess information is available from the corresponding author upon request.

## Abbreviations

aRR: Adjusted Relative Risk
BBW: Birth Weight
CAB: Community-Acquired Bacteremia
CDC: Centers for Disease Control and Prevention
CI: Confidence Interval
CRP: C-Reactive Protein
EMB: Eosin Methylene Blue
EOS: Early-Onset Sepsis
EUCAST: European Committee on Antimicrobial Susceptibility Testing
HABs: Hospital-Acquired Bacteremia
Hgb: Haemoglobin
LBW: Low Birth Weight
LMICs: Low- and Middle-Income Countries
LOS: Late-Onset Sepsis
NICUs: Neonatal Intensive Care Units
OR: Odds Ratio
PCR: Polymerase Chain Reaction
PPA: Postpartum Age
PPROM: Preterm Premature Rupture of Membranes
SGA: Small Gestational Age
WBC: White Blood Cell

## Supplementary material

The supplementary material for this article can be found at http://doi.org/10.1017/S0950268825100630.
