# Supplementary material for: ESBL-producing Klebsiella pneumoniae gut colonisation and subsequent health-care associated bacteraemia in preterm newborns: a descriptive cohort with nested case–control study
Source: Epidemiol Infect. 2025 Oct 6;153:e121. doi: 10.1017/S0950268825100630 (PMC12529433; doi:10.1017/S0950268825100630)
Supplement: Benboubker et al. supplementary material [file S0950268825100630sup001.zip › Supplementary Table A1.docx]

Table A1: clinical baseline, microbiological data, complications and outcomes of preterm neonates with *Kp*-ESBL bacteremia (32 cases)

| **Preterm number** | **Gender** | **PPA Hours** | **GA** | **Birth Weight (g)** | **Associated pathology** | **Delivery Mode** | **Premaurity level** | **NICU Stay** | **Apgar score** | **ESBL-KP carriage status and Genetic diversity** | **Time of bacteremia** | **Bacteremia biomarkers** | **Bacterimia ESBL-KP and Genetic diversity** | **Antimicrobial sensibility** | **Therapy** | **Clinical complications** | **Discharge** |
| --- | --- | --- | --- | --- | --- | --- | --- | --- | --- | --- | --- | --- | --- | --- | --- | --- | --- |
| P1 | F | 31 | 30 | 1200 | Hypothermia + Weak suck reflex | VB | Very preterm | 07 | 09  10 | CTXM1,  SHV,  TEM | D5 | CRP kinetic: 1 – 0- 48 – 51  WBC: 30000-15270-20570-12690  PLT: 143000-54000-3000-6000  Hgb: 16.8-12.6-7.2-11.5  NP : 10500-13600- | CTX-M1,  SHV, TEM | CRO, AK, COL CIP TIEN ERT | CRO  GN  TIEN  CIP  AK | Cerebral Hemorrhage+ Active Alveolar hemorrhage + Renal failure | Dead on Day 23 of life |
| P2 | F | 1 | 28 | 950 | Respiratory distress + Weak suck reflex | VB | Very preterm | 08 | 09 | CTXM1,  SHV,  NDM  OXA48 | D4 | CRP kinetic :88  WBC: 4790-5720  PLT: 4000/5000  Hgb: 16.9-9.6, NP : 3600-2940 | CTX-M1,  SHV, NDM | AK  CIP  COLI | CIP  AK | Sepsis and acute circulatory failure | Dead on Day 09 of life |
| P3 | F | 24 | 36 | 2500 | Respiratory Distress | VB | Moderate and late preterm | 9 | 10 | Control | D6 | CRP Kinetic: 77-2  WBC 16900/9700;  PLT: 44000/217000  Hgb: 14.8/18.1; | CTX-M1,  SHV, TEM | AK TIENCIP  LEVOF ERT | GN AK CRO  CIP | Active alveolar hemorrhage | Dead on Day 09 of life |
| P4 | M | 100 | 36 | 2400 | Respiratory Distress + Isolated esophageal onresia | VB | Moderate and late preterm | 12 | 10 | CTXM1, CTXM2  SHV, | D5 | CRP kinetic: 97/171/118/24  WBC 12000/7000/3000  PLT: 3000/18000/89000/171000  Hgb: 8.1 | CTX-M1,  SHV, TEM | AMC  AK  CIP  GN  IMI | GN  CRO  TIENCIP  AK  VAN | Pneumothorax | Dead on Day 10 of life |
| P5 | M | 6 | 34 | 2300 | Respiratory Distress | VB | Moderate and late preterm | 04 | 05  08 | Control | D3 | CRP kinetic :1-0-78 WBC 6 710  PLT: 32000/185000  Hgb: 12,8 Na+ à 135 | CTX-M1,  SHV, TEM | AK,CIP  COLI,ERT  TIEN,AN  NOR | AXY GN  AK  CRO  TIEN | Pneumothorax | Dead on Day 04 of life |
| P6 | F | 23 days | 37 | 2800 | Weak suck reflex | VB | Moderate and late preterm | 06 | 08  09 | Control | D3 | CRP kinetic: 109/150  Hgb: 14.1 WBC 26000/12350  PLT: 33000/238000  K 2.4 Na 130 | CTX-M1,  SHV, TEM | AK,CIP  COLI, TIEN,AN  ERT,NOR | AK-TIENCRO, CIP, | Urinary tract infection  Renal Failure | Desharge Day 07 of life |
| P7 | M | 6 | 30 | 1000 | Respiratory distress + Weak suck reflex | VB | Very preterm | 07 | 10 | Control | D4 | CRP kinetic: 121-13 -0  Hgb: à 13.3 / 21.3  WBC 4810/2300  PLT : 4000 /26000 / 117000  Ca++ 106, Prot T 32 | CTX-M1,  SHV, TEM | AK,CIP  COLI,  TIEN,AN  ERT,NOR | CRO, GN,  TIENAMXAK,  COLI, VO | Enterocolitis | Dead on Day 07 of life |
| P8 | M | 24 | 34 | 1500 | Respiratory distress + Weak suck | VB | Moderate and late preterm | 04 | 10 | Control | D2 | CRP kinetic :04 –80  Hgb: :20.1/19.3 WBC:3000/11710 PLT:140000/299000  LA KALIMIE A 5.5 | CTX-M1,  SHV, TEM | AK  GN  COLI  TIEN  ERT | GN, CRO, AK TIEN | Aggravation of respironory distress | Dead on Day 07 of life |
| P9 | M | 72 | 36 | 3100 | Respiratory distress | VB | Moderate and late preterm | 05 | 06  08 | Control | D3 | CRP kinetic: 1-38  Hgb: 15.8  WBC 9860/3460  PLT: 30000/146000 | CTX-M1,  SHV, TEM | AK,CIP  COLI,  TIEN,AN  ERT,NOR | GN  CROCIP  AK | Pneumothorax | Dead on Day 08 of life |
| P10 | F | 12 | 36 | 2200 | Respiratory distress | VB | Moderate and late preterm | 07 | 09  09 | CTXM1,  SHV,  TEM | D4 | CRP kinetic:6-61 Hgb: 12,9  WBC 5530/6580/24700  PLT: 100000/385000/312000  Na 115/ Kaliémie à 8.6/ calcémie à 1.68/ | CTX-M1,  SHV, TEM | AK,CIP  COLI,  TIEN,AN  ERT,NOR  SXT,CRO | GN  AK  CROTIEN | Anemiea  Active Alveolar Hemorrhage | Dead on Day 12 of life |
| P11 | F | 1 | 29 | 1200 | Respiratory distress | C/S | Very preterm | 12 | 09  08 | CTXM1, CTXM9  SHV, | D8 | CRP kinetic: 19/51/7/  WBC: 33400/2960/3300/5000  PLT: 20K/137K/126K/249K  Hgb: 16  NP : 23000/1700/1000/2700 | CTX-M1,  SHV, TEM | COL | TIEN  VAN  COLI | Renal Failure | Died on Day 13 of life |
| P12 | M | 76 | 34 | 1500 | Weak suck reflex | VB | Moderate and late preterm | 02 | 08  09 | Control | D2 | CRP kinetic: 0  WBC: 12900  PLT: 310k,Hgb: 15,NP: 6200 | CTX-M1,  SHV, TEM | COLI,  NOR,LEV  AK,PIP | AXY, GN | Good progress | Discharge on day 02 of life |
| P13 | M | 01 | 35 | 3600 | Acute fetal Distress | VB | Moderate and late preterm | 07 | 05  08 | CTXM1,  SHV, | D4 | CRP kinetic: 1 - 385 - 432 203 Hgb: 09/11.4  WBC 15460/1950/1360/6900  NP: 750  PLT: 12K/13K/211K, K+ 6.1 | CTX-M1,  SHV, TEM | AK, CIP  ERT, TIEN  NOF, SXT | GN, AK TIEN,  CRO, CIP,  VAN,  COLI | Aggravation of respiratory distress | Died on Day 08 of life |
| P14 | M | 72 | 37 | 2500 | Respiratory distress | VB | Moderate and late preterm | 09 | 10 | CTXM1,  SHV, | D7 | CRP kinetic: 77/0/2  Hgb: 14.8/18.1;  WBC 16900/9700;  PLT: 44000/217000; Na+=131 | CTX-M1,  SHV, TEM | AK  CIP  ERT  TIEN  NOFSXT | CRO, GN,  CIP, AMK | Pneumothorax  Active Alveolar hemorrhage | Died on Day 10 of life |
| P15 | M | 1 | 27 | 760 | Respiratory distress | VB | Extremely preterm | 03 | 10  10 | Control | D2 | CRP kinetic: missed  WBC: 2670  PLT: 74000, Hgb: NP : 100 | CTX-M1,  SHV, TEM | AK,CIP  COLI,ERT  GN,TIEN  NOR | CRO, GN, | Sepsis And Acute Circulatory Failure | Died on Day 03 of life |
| P16 | F | 48 | 36 | 3500 | Respiratory distress | VB | Moderate and late preterm | 47 | 07  09 | CTXM1,  SHV,  TEM |  | CRP kinetic: 0/1  WBC:13290/18000/23000  PLT: 446k/128k/121k/ | CTX-M1,  SHV, TEM | AK,CIP  COLI,ERT  GN,TIEN |  | Good Progress | Discharge on Day 47 of life |
| P17 | M | 30 | 36 | 3300 | Respiratory distress +generalized cyanosis | VB | Moderate and late preterm | 03 | 10 | Control | D2 | CRP kinetic :80-11-3  Hgb: 16.5;  WBC 17780;  PLT: 182000;  Ca2+=58 --> 68; | CTX-M1,  SHV, TEM | AK,CRO  CAZ,CIP  COLI,ERT  GN, TIEN  LEV, NOR | GEN, CRO, AK,  CIP | Aggravation of respiratory Distress | Died on Day 03 of life |
| P18 | F | 4 | 27 | 1560 | Respiratory distress  Extremely preterm | VB | Extremely preterm | 60 | 09  10 | CTXM1,  CTXM2  SHV,  TEM |  | CRP kinetic: 2/7/3/7  WBC: 8k/16k/23k  PLT: 324k/222k/318k  Hgb: 10.8/16 | CTX-M1,  SHV, TEM | AM,AMC  CRO,CIP  COLI,ERT  TIEN,  NOR, SXT | CRO  GN | Low Weight  Weight loss | Discharge on Day 60 of life |
| P19 | F | 36 | 36 | 2000 | Weak suck + premonurity | VH | Moderate and late preterm | 20 | 07  10 | CTXM1,  SHV, |  | CRP kinetic: 7/37/73/77/95/88/1  WBC: 10000/16000/8400/17000/6000, PLT: 23500/16000/ 4000/3000/34000/226000  NP: 4000/2000 | CTX-M1, CTX-M2, SHV, OXA-48 | TIEN  CIP  AK | TIEN  CIP  AK | Enterocolitis | Discharge on Day 20 of life |
| P20 | F | 1 | 30 | 1600 | Respiratory distress + Weak suck + premonurity | VB | Very preterm | 39 | 08  09 | CTXM1,  CTXM9  SHV,  TEM |  | CRP kinetic: 37/6.8/50/25/47/2  WBC: 13000/2300/14500  PLT: 87000/29000/7000/4000  NP : 9000/1000/8000 | CTX-M1,  SHV, | AK,CRO  CIP,COLI  ERT,TIEN  GN, SXT | CRO, GN  CIP, AK  COLI | Good progress | Discharge on Day 40 of life |
| P21 | F | 2 | 30 | 1800 | Respiratory distress + Weak suck + premonurity | VB | Very preterm | 06 | 07  10 | CTXM1,  SHV,  TEM | D4 | CRP kinetic: 33-28-26 Hgb: 14.6/15,8, WBC 10 380/4290  PLT: 50 000/201 000  K+ 6.6(sang hémolysé) | CTX-M1,  SHV, TEM | AK,CRO  CIP,COLI  ERT,TIEN  GN | CRO, GN,  AXY, AK,  TIEN | Aggravation of respiratory distress | Died on Day 07 of life |
| P22 | F | 1 | 28 | 940 | Respiratory distress + Weak suck reflex | VB | Very preterm | 08 | 08  08 | CTXM1,  SHV,  NDM  OXA48 | D4 | CRP kinetic :88  WBC: 4790-5721  PLT: 4000/5000  Hgb: 16.3-9.6, NP: 3600-2940 | CTX-M1,  SHV, NDM | AK, AMC CRO, CIP, COLI, ERT,TIEN | CIP, AK | Sepsis and acute circulatory failure  Active Alveolar Hemorrhage | Died on Day 09 of life |
| P23 | F | 24 | 36 | 2500 | Respiratory distress | C/S | Moderate and late preterm | 9 | 10  09 | Control | D6 | CRP Kinetic: 77-2  WBC 16900/9700;  PLT: 44000/217000  Hgb: 14.8/18.1; | CTX-M1,  SHV, TEM | AK, CIP, ERT,NOR,TIEN, LEV | CIP AK  CRO  GN | Aggravation of Respiratory Distress | Dead on Day 09 of life |
| P24 | M | 6 | 35 | 2300 | Respiratory distress + premonurity | VB | Moderate and late preterm | 04 | 05  08 | Control | D3 | CRP kinetic :1-0-78 WBC 6 710  PLT: 32000/185000  Hgb: 12,8 Na+ à 135 | CTX-M1,  SHV, TEM | AK,CIP  COLI,ERT  TIEN,  NOR | AXY, GN,  AK,  CRO, TIEN | Aggravation of Respiratory Distress | Dead on Day 05 of life |
| P25 | M | 6 | 30 | 1200 | Respiratory distress + Weak suck reflex + premonurity | VB | Very preterm | 07 | 10  09 | CTXM1,  SHV,  TEM | D4 | CRP kinetic: 121-13 -0  Hgb: à 13.8 / 21.3  WBC 4810/2300  PLT : 4000 /26000 / 117000  Ca++ 106, Prot T 32 | CTX-M1,  SHV, | AK,CIP  COLI,  TIEN,ERT  NOR | CRO, GN, TIENAM, AK, COLI, VAN | Active Alveolar Hemorrhage | Dead on Day 07 of life |
| P26 | M | 76 | 34 | 1450 | Weak suck reflex + premonurity | VB | Moderate and late preterm | 02 |  | Control | D2 | CRP kinetic: 0  WBC: 13900, PLT: 310k  Hgb: 15, NP: 6200 | CTX-M1,  SHV, TEM | COLI  NOR,LEV,AK PIP | AXY, GN | Good Progress | Desharge on 02day of life |
| P27 | M | 72 | 37 | 2550 | Respiratory distress + premonurity | VB | Moderate and late preterm | 09 | 10  10 | CTXM1,  SHV, | D7 | CRP kinetic: 77/0/2  Hgb: 14.8/18.8;  WBC 16900/9700;  PLT: 44000/217000; Na+=131 | CTX-M1,  SHV, | AK, CIP  ERT, TIEN  NOR, SXT | CRO, GN, CIP, AMK | Active Alveolar hemorrhage  Pneumothorax | Dead on Day 10 of life |
| P28 | M | 1 | 27 | 760 | Respiratory distress + premonurity | VB | Extremely preterm | 03 | 10  10 | Control | D2 | CRP kinetic: Missed  WBC: 2680  PLT: 75000  NP : 100 | CTX-M1,  SHV, | AMK,CIP  COLI,ERT  GN,TIEN  NOR | CRO, GN, | Aggravation of Respiratory Distress | Dead on Day 03 of life |
| P29 | F | J35 | 36 | 3500 | Respiratory distress | VB | Moderate and late preterm | 47 | 07  09 | CTXM1,  SHV,  TEM |  | CRP kinetic: 0/1  WBC:13290/18400/23000  PLT: 446k/128k/121k/ | CTX-M1,  SHV, | AK,CIP  COLI,ERT  GN,TIEN  NOR |  | Good progress | Discharge on Day 47 of life |
| P30 | M | 30 | 36 | 2300 | Respiratory distress +generalized cyanosis | VB | Moderate and late preterm | 03 | 10  10 | Control | D2 | CRP kinetic :89-14-3  Hgb: 16.5;  WBC 17880;  PLT: 182000;  Ca2+=58 --> 68; | CTX-M1,  SHV, | AK,CRO  CAZ,CIP  COLI,ERT  GN, TIEN  LEV,NOR | GEN, CRO, AK  CIP | Aggravation of respiratory distress | Dead on Day 03 of life |
| P31 | F | 4 | 27 | 1560 | Respiratory distress | VB | Extremely preterm | 60 | 09  10 | CTXM1,  SHV,  TEM |  | CRP kinetic: 2/7/4/7  WBC: 8k/16k/23k  PLT: 324k/222k/318k  Hgb: 10.8/16 | CTX-M1,  SHV, | AM,AMC  AMP,  CRO,CIP  COLI,ERT  TIEN | CRO  GN | Good Progress  low weight | Discharge on 60 Day of life |
| P32 | F | 1 | 30 | 1600 | Respiratory distress | VB | Very preterm | 39 | 08  09 | CTXM1,  SHV,  TEM |  | CRP kinetic: 37/6.8/55/25/47/2  WBC: 14000/2300/14500  PLT: 87000/29000/7000/4000  NP: 9000/1000/8000 | CTX-M1,  SHV, TEM | AK,CRO  CIP,COL  ERT,TIEN  GN, SXT | CRO, GN  CIP, AK  COLI | Good Progress | Discharge on 40 Day of life |

WBC: White Blood Cell Count, CRP: C-Reactive Protein, PLT: Plonelet count; Hgb: Hemoglobin, NP: Neutrophil Percentage, PT: Prothrombin Time

IMP, imipenem; MEM, meropenem;TIEN,tienam; ERT, ertapenem; PIP/TZ, piperacillin-tazobactam; TIC/AC, ticarcilline-acide clavulanique; AMC, amoxicilline-acide clavulanique; AM, amoxicilline; CAZ, ceftazidime; FEP, cefepime; CRO, ceftriaxone; CTX, cefotaxime; AK, amikacin; GN, gentamicin; CIP, ciprofloxacin; LEV, levofloxacin; NA, nalidixic acid; SXT, trimethoprim/sulfamethoxazole; FOT, fosfomycin; COL,colymicin; AXY,axymicin; NOR: norfloxacin ;VAN:vancomycin

CR-*Kp,* carbapenem-resistant Klebsiella pneumoniae; NDM, New Delhi metallo-β-lactamase; OXA-48, oxacillinase 48; CTX-M1-2-9, Cefotaximase-Munich; SHV, sulfhydryl variable; TEM, temonera

C/S = Cesarean Section; VB= Vaginal Birth; CRP = C-Reactive Protein; GA = Gestonional Age; Hgb = Hemoglobin; LONS = lone-onset neononal sepsis; PLT = Plonelet; PPA = Postpartum Age; WBC = White Blood Count
