# Supplementary material for: ESBL-producing Klebsiella pneumoniae gut colonisation and subsequent health-care associated bacteraemia in preterm newborns: a descriptive cohort with nested case–control study
Source: Epidemiol Infect. 2025 Oct 6;153:e121. doi: 10.1017/S0950268825100630 (PMC12529433; doi:10.1017/S0950268825100630)
Supplement: Benboubker et al. supplementary material [file S0950268825100630sup001.zip › Supplementary Table A2.docx]

| **Antibiotic** | **Dosage and Frequency for Preterm Neonates** |
| --- | --- |
| Tienam | **General Dosage:** The typical dosage for neonates is 25-40 mg/kg/day of imipenem, divided into multiple doses.  **Frequency:**  - For neonates less than 7 days old: Every 12 hours.  - For neonates older than 7 days: Every 8 hours. |
| Ciprofloxacin | **Standard Dosage:**10 mg/kg/dose administered intravenously every 12 hours is commonly recommended for treating serious infections in preterm neonates.  For more severe infections, particularly those caused by resistant organisms such as Pseudomonas aeruginosa, a higher dosage of 20 mg/kg/dose may be considered, administered every 12 hours |
| Amiklin | **General Recommendation**: a standard dose of 15 mg/kg/day for preterm infants, which can be divided into multiple doses based on clinical circumstances.  **Specific Regimens:**  **For infants ≤29 weeks gestation**:   - 0–7 days old: 14 mg/kg every 48 hours. - 8–28 days old: 12 mg/kg every 36 hours.   **For infants ≥30 weeks gestation:**   - 0–7 days old: 12 mg/kg every 36 hours. - ≥8 days old: 12 mg/kg every 24 hours. |
| Ceftriaxone (Triaxon) | **Standard Dosage:** For neonates, the typical dose is 50-100 mg/kg/day, divided into one or two doses. The specific dosing may vary based on the severity of the infection and the pathogen involved. |
| Gentamicin | **Preterm Infants (< 30 weeks gestation):** 3 mg/kg administered every 36 hours.  **Infants 30 to 34 weeks gestation:** 4 mg/kg administered every 24 hours.  **Term Newborns (> 35 weeks gestation):** 4 mg/kg administered every 24 hours**.** |
| Colymicin (Colistin) | **Loading Dose:** Start with 10,000 to 20,000 IU/kg/day, divided into multiple doses.  **Maintenance Dose:** Adjust based on renal function and clinical response; generally, around 5,000 to 10,000 IU/kg/day divided into two or three doses. |
| Vancomycin | **For preterm infants (<29 weeks gestation):** a common regimen is 15 mg/kg every 12 hours.  **For infants with higher postmenstrual ages (30-44 weeks):** dosing can vary:   - 20 mg/kg every 24 hours for those with renal impairment. - Adjustments may be made based on serum creatinine levels and clinical response. |

Table A2: Antimicrobial therapy protocol
