# Supplementary figures and images for: ESBL-producing Klebsiella pneumoniae gut colonisation and subsequent health-care associated bacteraemia in preterm newborns: a descriptive cohort with nested case–control study
Source: Epidemiol Infect. 2025 Oct 6;153:e121. doi: 10.1017/S0950268825100630 (PMC12529433; doi:10.1017/S0950268825100630)

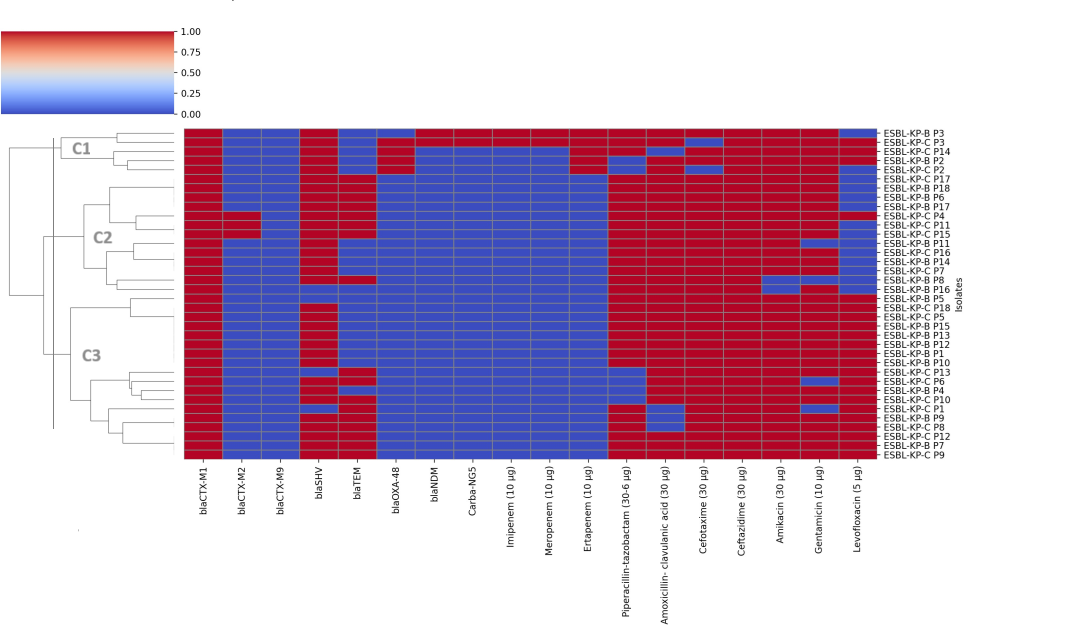

Supplement: Benboubker et al. supplementary material [file S0950268825100630sup001.zip › Figure A1.png]
